# Vaccination against Human Influenza A/H3N2 Virus Prevents the Induction of Heterosubtypic Immunity against Lethal Infection with Avian Influenza A/H5N1 Virus

**DOI:** 10.1371/journal.pone.0005538

**Published:** 2009-05-14

**Authors:** Rogier Bodewes, Joost H. C. M. Kreijtz, Chantal Baas, Martina M. Geelhoed-Mieras, Gerrie de Mutsert, Geert van Amerongen, Judith M. A. van den Brand, Ron A. M. Fouchier, Albert D. M. E. Osterhaus, Guus F. Rimmelzwaan

**Affiliations:** Department of Virology, Erasmus Medical Center, Rotterdam, The Netherlands; University of Pretoria, South Africa

## Abstract

Annual vaccination against seasonal influenza viruses is recommended for certain individuals that have a high risk for complications resulting from infection with these viruses. Recently it was recommended in a number of countries including the USA to vaccinate all healthy children between 6 and 59 months of age as well. However, vaccination of immunologically naïve subjects against seasonal influenza may prevent the induction of heterosubtypic immunity against potentially pandemic strains of an alternative subtype, otherwise induced by infection with the seasonal strains.

Here we show in a mouse model that the induction of protective heterosubtypic immunity by infection with a human A/H3N2 influenza virus is prevented by effective vaccination against the A/H3N2 strain. Consequently, vaccinated mice were no longer protected against a lethal infection with an avian A/H5N1 influenza virus. As a result H3N2-vaccinated mice continued to loose body weight after A/H5N1 infection, had 100-fold higher lung virus titers on day 7 post infection and more severe histopathological changes than mice that were not protected by vaccination against A/H3N2 influenza.

The lack of protection correlated with reduced virus-specific CD8+ T cell responses after A/H5N1 virus challenge infection. These findings may have implications for the general recommendation to vaccinate all healthy children against seasonal influenza in the light of the current pandemic threat caused by highly pathogenic avian A/H5N1 influenza viruses.

## Introduction

Since 2003, more than 380 human cases of infection with highly pathogenic avian influenza A virus (IAV) of the H5N1 subtype have been reported to the World Health Organization (WHO) of which more than 60% were fatal [1]. Because of the continuous spread of these viruses among domestic birds, the frequent introduction into wild birds and the increasing number of human cases, a pandemic outbreak caused by influenza A/H5N1 viruses is feared [2]–[4].

It has been demonstrated in animal models that prior exposure to an IAV can induce heterosubtypic immunity to infection with an IAV of an unrelated subtype (for review see [5]). Also in humans there is evidence that infection with IAV can induce heterosubtypic immunity [6]. Individuals that had experienced an infection with an H1N1 IAV before 1957 less likely developed influenza during the H2N2 pandemic of 1957 [6]. In particular, the induction of cell-mediated immune responses after infection contributes to protective immunity against infection with heterosubtypic IAVs. The presence of cross-reactive cytotoxic T lymphocytes (CTL) in humans inversely correlated with the amount of viral shedding in the absence of antibodies directed against the virus used for experimental infection [7]. It is well documented that seasonal human IAVs and avian IAVs share CTL epitopes located in the internal viral proteins like the nucleoprotein [8]–[10]. Thus, cell-mediated immunity induced by natural infection with seasonal IAVs may confer protection against heterosubtypic pandemic influenza viruses. In this respect, the disproportional age distribution of severe human H5N1 cases is of interest [11]. Especially younger individuals are at risk and although other confounding factors cannot be excluded, it is tempting to speculate that young subjects have been infected with seasonal influenza viruses less frequently and therefore have not developed protective heterosubtypic immune responses against infection with the highly pathogenic avian A/H5N1 viruses.

Since seasonal IAVs of the H3N2 and H1N1 subtypes cause epidemic outbreaks annually associated with excess morbidity and mortality mainly among infants, the elderly, immuno-compromised and other high-risk patients, influenza vaccination is recommended for these high-risk groups. In general, the influenza vaccines most frequently used are inactivated vaccines, including subunit preparations that consist of the viral hemagglutinin (HA) and neuraminidase (NA). Due to the higher risk of complications and hospitalizations secondary to influenza in children [12], [13], annual vaccination of all healthy children 6 to 59 months of age was recommended in various countries including the United States since 2007 [14].

However, annual vaccination may prevent the induction of heterosubtypic immunity by infection with seasonal influenza virus strains. In addition, it is unlikely that seasonal inactivated influenza vaccines, unlike live attenuated vaccines, induce heterosubtypic immunity since they induce cross-reactive CTL responses inefficiently [15], [16].

Thus, we hypothesized that vaccination against seasonal flu prevents the induction of cross-protective cell-mediated immunity, which consequently may lead to more severe clinical outcome of infection with a future pandemic virus. Here we show in a mouse model that protective immunity against lethal infection with H5N1 IAV Indonesia/5/05 (IND/05) was induced by infection with H3N2 IAV HongKong/2/68 (HK/68), which was prevented by effective vaccination against the A/H3N2 virus. The lack of protection against IAV IND/05 correlated with reduced virus-specific CTL responses.

## Results

### Antibody responses against IAV HK/68 (H3N2) after vaccination

**Figure 1:**
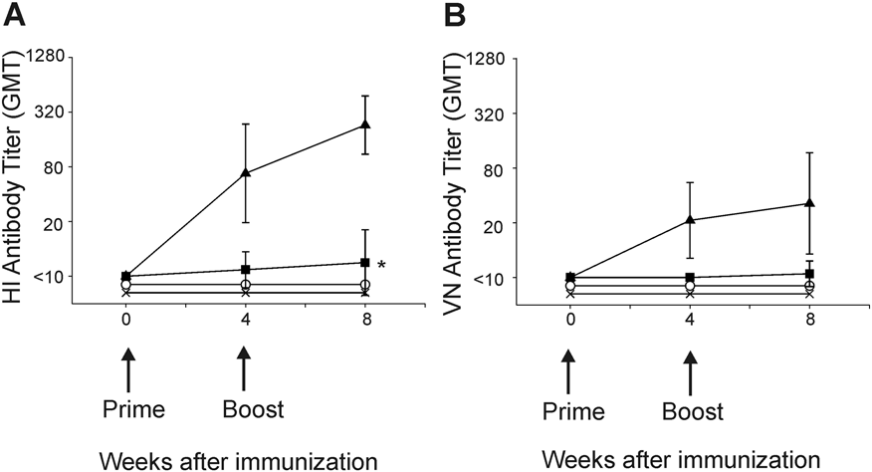
Induction of serum antibodies against IAV HK/68 (H3N2) by vaccination. Serum antibody levels were determined before and at the indicated time points after vaccination of mice with PBS (groups 1, 3 and 4; ○), subunit vaccine with alum (groups 2 and 5; ▴), subunit vaccine only (group 6; ▪) and alum only (group 7; ×) by HI assay (A) and VN assay (B).

Mice were vaccinated with subunit vaccine with or without Alum or were ‘mock’ vaccinated (**table 1**). HI antibody titers were detected 28 days after the first vaccination with subunit and Alum (groups 2 and 5) and in 3 out of 26 mice vaccinated with unadjuvanted subunit vaccine (group 6). Four weeks after the second vaccination, geometric mean titers (GMTs) increased to 244 and 218 in mice from group 2 and group 5, respectively. Four mice of group 6 developed detectable HI-antibody responses with a GMT of 48, the other mice of this group did not seroconvert (**figure 1A**). Sera of mice were also analysed for the presence of virus neutralizing (VN) antibodies. Four weeks after the second vaccination, mice vaccinated with adjuvanted subunit vaccine developed VN antibodies with a GMT of 38 and 29 in group 2 and group 5 respectively, while only two mice of group 6 developed detectable VN antibody titers (**figure 1B**).

**Table 1:** Experimental groups and design of the study.

Experimental group	Vaccination		Infection	
	Subunit	Adjuvant	HK/68	IND/05
1	−	−	−	−
2	+	+	+	+
3	−	−	+	+
4	−	−	−	+
5	+	+	−	+
6	+	−	+	+
7	−	+	+	+

Mice were divided over seven groups and were either vaccinated twice with subunit vaccine with or without adjuvant (Alum), PBS, or adjuvant only as indicated. Four weeks after the second vaccination, mice were infected with IAV HK/68 (H3N2) or mock-infected. Twenty-nine days after the infection with IAV HK/68, mice were challenged with IAV IND/05 (H5N1).

### Outcome of infection with IAV HK/68 (H3N2)

**Figure 2:**
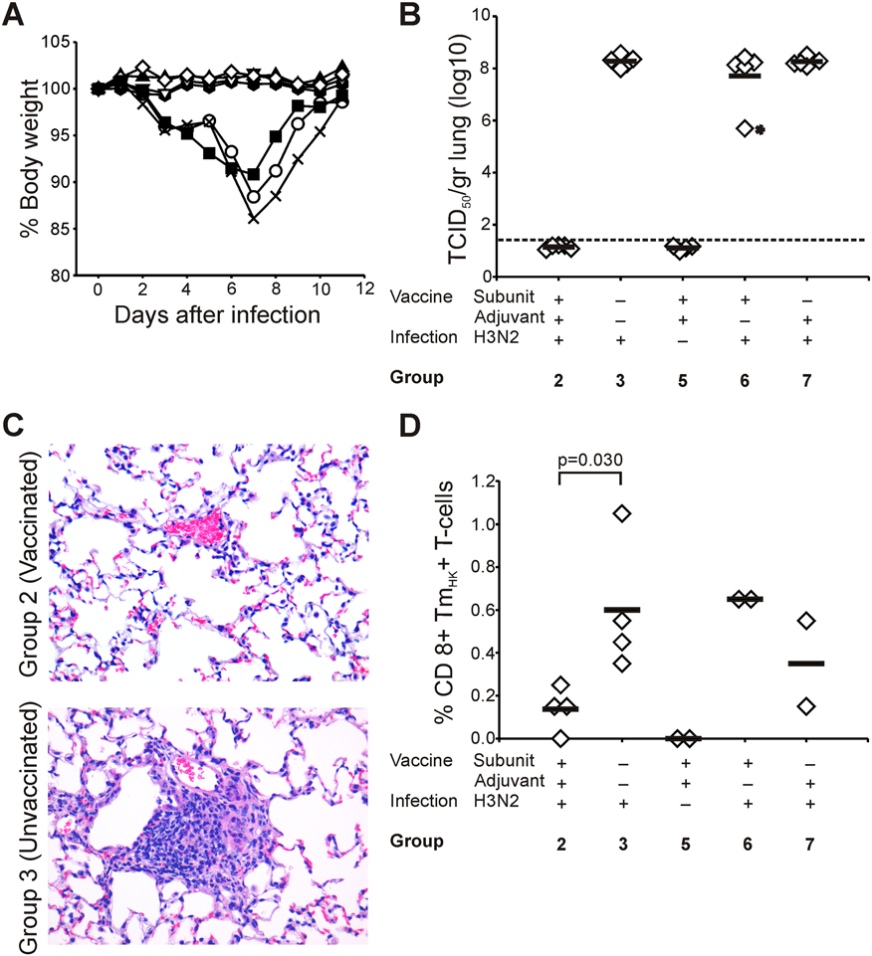
Outcome of infection with IAV HK/68 (H3N2). Mice were inoculated with IAV HK/68 (groups 2 (▴), 3 (○), 6 (▪) and 7 (×)) or PBS (groups 1 (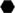), 4 (▿) and 5 (⋄)). (A) Body weight after infection was determined daily and expressed as the percentage of the original body weight before infection. (B) Lung virus titers measured on day 4 p.i. in mice from the indicated experimental groups. Horizontal bars represent the average titers of five mice. The dotted line represents the cut-off value for obtaining a positive result. *This mouse from group 6 had before infection an HI antibody titer of 40. (C) Vaccination prevented the induction of iBALT after infection. Twenty-eight days post infection with IAV HK/68 iBALT was detected in mice from group 3, but not in mice from group 2. Lung tissue sections were stained with HE. (D) Virus-specific CD8+ T cell responses detected 28 days post infection. Splenocytes of mice from the indicated experimental groups were tested for the presence of CD8+ T cells that bound the H2-Db NP_HK_ Tetramer. Horizontal bars represent the average of 2–4 mice. The difference in %CD8+ Tm+ T cells between groups 2 and 3 was statistically significant (*P* = 0.030).

Mice that developed HI-antibodies against IAV HK/68 (all mice of group 2 and four of group 6) were protected from weight loss after infection with IAV HK/68, while mice of other groups lost weight until day seven post infection (p.i.) and showed mild clinical symptoms for 2–3 days (**figure 2A**). Clinical signs and weight loss after infection correlated well with virus titers in the lungs of infected mice 4 days p.i.. No virus was detected in lungs of mice vaccinated with adjuvanted subunit vaccine, while the average lung virus titer of mock-vaccinated mice was 10^8.1^ TCID_50_/gram lung. Similar titers were observed for the mice in groups 6 and 7 with the exception of one mouse in group 6 with a HI antibody titer of 40 induced by vaccination with unadjuvanted subunits that had a lung virus titer of 10^5.7^ TCID_50_/gram lung (**figure 2B**). The virus titers detected on day 4 p.i. correlated with the absence or presence of virus infected cells in the lungs detected by immunohistochemistry (data not shown).

### Virus-specific CTL and antibody responses after infection with IAV HK/68 (H3N2)

Four days p.i. with IAV HK/68 the frequency of splenic CD8+ T lymphocytes specific for the NP_366–374_ epitope of IAV HK/68 (CD8+ Tm_HK_+ T-cells) as determined by tetramer staining remained at background levels in all groups (data not shown).

In all infected mice a raise in the frequency of CD8+ Tm_HK_+ T-cells was detected twelve days p.i.. No statistically significant differences were observed between the experimental groups. Essentially the same results were observed using intracellular IFN-γ staining after re-stimulation with peptides representing the NP_366–274_ and PA_224–233_ epitopes of IAV HK/68 (NP_HK_ and PA_HK_). The NP_HK_ and PA_HK_ specific CTL induced by infection with IAV HK/68 cross-reacted to various extents with their counterparts derived from IAV IND/05 (NP_IND_ and PA_IND_). The cross-reactive nature of a proportion of the NP_366–374_ specific CTL was confirmed by double staining with Tm_HK_ and Tm_IND_ (data not shown).

By day 28 p.i. with IAV HK/68, just before challenge infection with IAV IND/05, the frequency of virus-specific CTL in the spleen had declined and virus-specific CTL were not detectable by intracellular IFN-γ staining. However, Tm_HK_ and Tm_IND_ positive cells were detected in mice that were mock vaccinated prior to infection (group 3). Strikingly, the frequency of Tm_HK_ positive CD8+ T lymphocytes was significantly lower in mice of group 2 that were effectively vaccinated against infection with IAV HK/68 (*p* = 0.030) (**figure 2D**).

### Vaccination prevents induction of iBALT after IAV HK/68 infection

Following infection with IAV HK/68, no significant lesions were found in lungs of mice vaccinated with adjuvanted subunit vaccine (group 2), whereas mice that were mock-vaccinated or vaccinated with Alum or subunit preparation only (mice of groups 3, 6 and 7) developed a multifocal mild subacute necrotizing bronchopneumonia four days after infection, which on day 12 p.i. progressed into a multifocal moderate chronic necrotizing bronchopneumonia. On day 28 p.i., these mice had developed perivascular moderate proliferation of inducible Bronchus Associated Lymphoid Tissue (iBALT), consisting mainly of mononuclear cells, which was absent in mice effectively vaccinated against infection with IAV HK/68 (**figure 2C**).

### Effective vaccination prevents heterosubtypic immunity against IAV IND/05 (H5N1)

**Figure 3:**
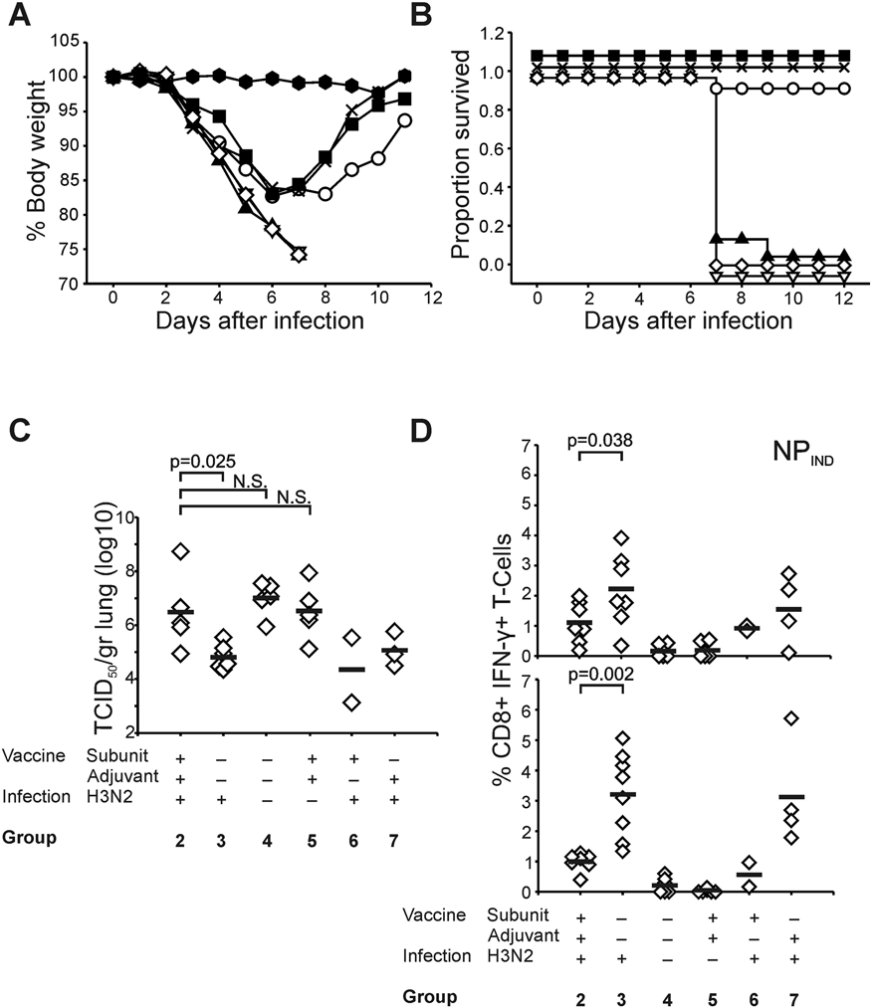
Outcome of infection with IAV IND/05 (H5N1). Mice were inoculated with IAV IND/05 (groups 2 (▴), 3 (○), 4 (▿), 5 (⋄), 6 (▪) and 7 (×)) or PBS (group 1 (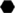). (A) Body weight after infection was determined daily and expressed as the percentage of the original body weight before infection. (B) Survival rates after infection with IAV IND/05. The proportion of mice from the indicated groups that survived infection is shown in a Kaplan-Meier plot. Moribund animals were euthanized when they reached pre-fixed criteria regarding weight loss (>20%) and disease severity score, which was used to determine mortality rates. (C) Lung virus titers measured on 7 days p.i. in mice from the indicated groups. Horizontal bars represent the average of 2–6 mice. The difference in virus titers between mice of group 2 and group 3 was statistically significant (p = 0.025). N.S.: not significant. (D) Virus-specific CD8+ T cell responses on day 7 p.i.. The frequency of CD3+ CD8+ splenocytes specific for peptide NP_366–374_ and PA_224–233_ derived from IAV IND/05 was determined by intracellular IFN-γ staining. The horizontal bars represent the average frequency of IFN-γ+ cells in the CD8+ T cell population of 2–7 mice in the indicated groups. Differences between group 2 and group 3 were statistically significant for both peptides.

After infection with IAV IND/05, all mice developed clinical signs (weight loss, ruffled fur, lethargy) from day two p.i. onwards. Mice that developed clinical signs p.i. with IAV HK/68 (groups 3, 6 and 7) lost weight until day 6–7 after infection with IAV IND/05 and then started to gain weight and fully recovered, while mice of other groups, not previously infected with IAV HK/68 (groups 4 and 5) and more strikingly, those effectively vaccinated against infection with IAV HK/68 (group 2) lost significantly more weight (group 2 versus group 3: *p* = 0.0001) on day 7 p.i. with IAV IND/05 and showed more severe clinical signs (lethargy, ruffled fur, hunched posture) than mice of the other groups (**figure 3A**). Moribund animals were euthanised when they reached pre-fixed criteria regarding weight loss (>20%) and clinical signs, which was used to determine mortality rates. One mouse out of 10 (10%) of group 2 survived lethal challenge, while all mice but one (91%) of group 3 survived lethal challenge (n = 11). This difference in survival rate was statistically significant (*p* = 0.0003) as was calculated with the Logrank test (**figure 3B**). All other mice not previously exposed to IAV HK/68 became moribund, whereas all mice not adequately vaccinated against IAV HK/68 (groups 6 and 7) survived.

### Replication of IAV IND/05 (H5N1) in the lungs

The lung virus titers at days four and seven p.i. were compared between groups of IAV IND/05 infected mice. Four days p.i. no significant differences were found between mice of different groups. The average virus titer in mice of group 3 was 10^7.7^ TCID_50_/gram lung, which was similar to that observed in mice from group 2 that were effectively vaccinated against IAV HK/68 (10^7.6^ TCID_50_/gram lung). In contrast, there were significant differences in lung viral titers between mice of the different groups seven days p.i. (**figure 3C**). Group 3 mice, not vaccinated against infection with IAV HK/68, had virus titers of 10^4.8^ TCID_50_/gram lung while mice of group 2, vaccinated with adjuvanted subunits, had significantly higher virus titers with an average of 10^6.5^ (*p* = 0.025), which was similar to that observed in naïve mice infected with IAV IND/05 virus (group 4) or those that were vaccinated against, but not infected with IAV HK/68 virus (group 5). Mice unsuccessfully vaccinated against IAV HK/68 infection with adjuvant or subunits only also displayed lower lung viral titers (groups 6 and 7).

### Induction of CD8+ T cell responses p.i. with IAV IND/05 (H5N1)

Four and seven days p.i. infection with IAV IND/05, splenocytes were stained for intracellular IFN-γ after incubation with peptides NP_IND_ and PA_IND_. Four days p.i., no virus-specific CD8+ T cell responses were detected in any of the IAV IND/05 infected mice. However, seven days p.i, anamnestic NP_IND_ and PA_IND_ specific IFN-γ+CD8+ T-cell responses were observed in mice from group 3, which were significantly lower in mice effectively vaccinated against IAV HK/68 (group 2) (*p* = 0.038 and *p* = 0.002 respectively) (**figure 3D**)

### Histopathology and detection of infected cells after infection with IAV IND/05 (H5N1)

On day four p.i. with IAV IND/05, mice developed a multifocal severe subacute necrotizing bronchopneumonia, of which the severity was similar for all experimental groups. However, seven days p.i. there were marked differences between the groups. The mock-vaccinated mice or those vaccinated with adjuvant only prior to infection with IAV HK/68 had a multifocal moderate chronic necrotizing bronchopneumonia characterized by a perivascular core of lymphocytes and plasma cells, proliferation of bronchiolar epithelium and hyperplasia of pneumocytes with a type II appearance. In contrast, mice of groups 4, 5 and especially group 2 had more severe lung pathology characterized by a multifocal to coalescing severe subacute necrotizing bronchopneumonia.

**Figure 4:**
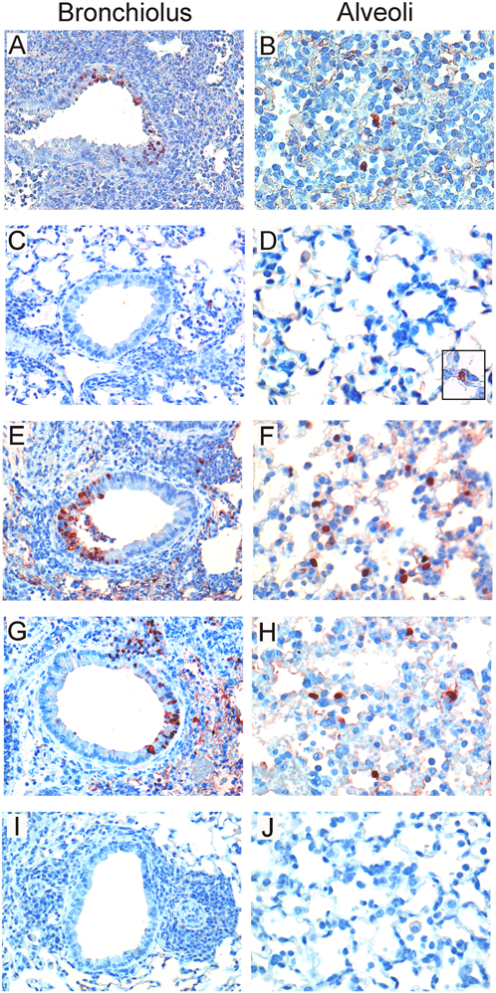
Histopathological analysis and immunohistochemistry of the lungs of mice infected with IAV IND/05. Mouse lung sections were stained for influenza A virus nucleoprotein. Cytoplasm of infected cells stain red, the nuclei of infected cells stain deep red. In the groups without a history of productive A/H3N2 infection, including group 2 (A,B), infection with IAV IND/05 led to severe histopathological changes and to viral antigen expression in cells of the bronchiolar walls and in the alveoli (group 4: E,F and group 5: G,H). In mice of groups 3 (C,D) and 7 (I,J) that had experienced a productive infection with IAV HK/68 only moderate histopathological changes were observed and virus infected cells were detected sporadically (see insert in panel D). For more information please see text.

In general, the extent of lung histopathology and the lung virus titers after infection with IAV IND/05 correlated with the presence of virus-infected cells in the lungs as determined by immunohistochemistry. Four days p.i., virus-infected cells were detected in all IAV IND/05 infected mice. In contrast, seven days p.i., antigen positive cells were found sporadically in lungs of mice of groups 3 (**figures 4C–D**) and 7 (**figures 4I–J**), whereas in the lungs of mice from group 2 (**figures 4A–B**), 4 (**figures 4E–F**) and 5 (**figures 4G–H**) virus-infected cells were still abundantly present.

## Discussion

Here we demonstrate that successful vaccination of mice against human IAV HK/68 (H3N2) prevented the induction of heterosubtypic immunity against a lethal challenge with IAV IND/05 (H5N1). As a result, H3N2 vaccinated mice had a fatal clinical outcome of infection with IAV IND/05, associated with higher virus titers and more severe histopathological lesions in the lungs seven days p.i. and reduced virus-specific CD8+ T cell responses compared to mice that experienced a productive, self-limiting infection with IAV HK/68.

It has been well established that infection with IAV can induce a certain degree of protective immunity against infection with an heterosubtypic strain of IAV, which was already recognized more than 40 years ago [17]. This so-called heterosubtypic immunity was not only demonstrated in animal models [17]–[20] but there is also direct and indirect evidence that it exists in humans [6], [7] and that cell-mediated immune responses contribute to this type of immunity (for review see [21]).

Of special interest in this respect is that there is a disproportionate age distribution of human cases [11]. Especially younger subjects are at risk for severe A/H5N1 disease and fatal outcome, which may inversely correlate with the history of infections with seasonal influenza viruses and the cross-reactive CTL responses [8] associated with these infections. Also the results from the Cleveland study indicate that a prior infection with seasonal influenza virus strains induced protective immunity against a new heterosubtypic pandemic strain [6]. Nevertheless, severe A/H5N1 infections with fatal outcome do occur. However, little is known about the history of previous infections of these patients. Although most adults must have experienced an infection with seasonal influenza viruses, it is possible that individual cases did not develop adequate heterosubtypic immunity against A/H5N1 strains.

To test the hypothesis that successful immunization against seasonal influenza could interfere with the induction of heterosubtypic immunity, mice were vaccinated with an Alum-adjuvanted subunit vaccine. The use of an adjuvant was necessary since vaccination with subunit alone induced detectable antibody responses in a small proportion of mice only and would not provide a useful model for successful vaccination against seasonal influenza. Indeed, all mice vaccinated with Alum alone and most mice vaccinated with subunits alone were not protected against infection with A/H3N2 virus. In contrast, all mice vaccinated with adjuvanted subunits, were fully protected against infection with IAV HK/68. This prevented the induction of heterosubtypic immunity against infection with IAV IND/05 normally seen in mice that had experienced a productive IAV HK/68 infection. The severity of the clinical signs and histopathological lesions, the extent of weight loss, lung virus titers and mortality rates of these mice was comparable of those that were immunologically naïve prior to infection with IAV IND/05 (group 4) or that were vaccinated against IAV HK/68 virus, but not subsequently infected with IAV HK/68 virus (group 5). It could be argued that in the present study the vaccine matched the A/H3N2 virus perfectly, while under field conditions the match may not always be optimal allowing sub-clinical infections to occur, which may induce heterosubtypic immunity despite vaccination. However, also in our mouse model there is indication that in vaccinated mice sub-clinical infection with influenza virus A/HK/2/68 took place, since weak, short-lived virus-specific CTL responses were observed, which did not protect against challenge infection with the A/H5N1 strain.

Four weeks after infection with IAV HK/68 virus, the number of virus-specific CD8+ T cells in the spleen was significantly lower in mice vaccinated against IAV HK/68 than in unvaccinated mice. The differences were not observed at earlier time points p.i.. Further evaluation of the CD8+ Tm_HK_+ T cells indicated that the numbers of CD62L_high_ and CD127+ cells were higher in unvaccinated mice than in vaccinated mice on day 28 p.i. (data not shown). This may indicate that the control of IAV HK/68 replication in the lungs had prevented the efficient induction of virus-specific central and effector memory CD8+ T cell responses. These results resemble those found in a mouse model for *Listeria monocytogenes* infection, in which shortening of the duration of the infectious period did not impact the size of the primary CD8+ T cell response, but diminished the memory population of CD8+ T cells [22]. The analysis of the CD8+ T cells responses seven days after challenge infection with IAV IND/05 further indicated that indeed prior vaccination against HK/68 (H3N2) prevented the efficient induction of memory CTL responses. Both the secondary response to the NP_IND_ and the PA_IND_ epitope were reduced compared to the responses observed in un-vaccinated mice. Although it has been described that the NP_366–374_ is more immunodominant than the PA_224–233_ epitope in secondary CTL responses [23], a stronger response was observed against the PA_224–233_ epitope after infection with IAV IND/05. This could be explained by the lower cross-reactivity of CTL directed to the NP_366–374_ epitope derived from IAV HK/68 (ASNENMDAM) with that derived from IAV IND/05 virus (ASNENMEVM) compared to the cross-reactivity of CTL specific for the PA_224–233_ epitope as was observed after the analysis of the CTL measured by tetramerstaining p.i. with IAV HK/68 and IND/05 (data not shown). Apart from systemic CTL responses measured in the spleen also local CTL responses may contribute to protective immune responses, such as in the draining lymph nodes and in the lung tissue itself [24], [25]. Since the frequency of virus-specific CD8+ T cells in the spleen reflected that in the lymph nodes [26], [27], we analyzed CTL responses in the spleen only. It was of interest to note that infection with IAV HK/68 resulted in the formation of iBALT structures. Prior vaccination against IAV HK/68 infection prevented the formation of iBALT completely. iBALT consists mainly of B cells, T cells and dendritic cells and it has been shown that mice with iBALT but without peripheral lymphoid organs can clear virus infection [28]. Also in humans, T cells specific for viral respiratory pathogens have been detected in lung tissue and may play a protective role against subsequent infections in this species as well [29]. Although no IAV IND/05 cross-reactive antibodies were detected by VN or HI assay on the day of challenge infection, it is possible that infection with IAV HK/68 induced M2 specific antibodies that potentially cross-reacted with the M2 protein of IAV IND/05. However it is unlikely that these antibodies accounted for the heterosubtypic immunity induced by primary infection with IAV HK/68 [30], [31].

Thus prior infection with seasonal influenza viruses, which generally results in a self-limiting upper respiratory tract infection, may afford at least partial protection against potentially pandemic heterosubtypic influenza virus strains. At present vaccination against seasonal influenza is recommended for all healthy children 6–59 months of age in a number of countries, including the USA [14]. Also in Europe vaccination of children is currently considered and a number of countries already decided to recommend vaccination of healthy children [32]. Although vaccination is (cost-) effective in this age group [33]–[37], it may interfere with the induction of heterosubtypic immunity against potentially pandemic strains of a novel subtype, e.g. H5N1, by creating an immunological “blind spot”. Furthermore, the use of adjuvants is considered to increase vaccine efficacy in young children [38]. Thus during a next pandemic, especially children that received the annual flu-shot would be at higher risk to develop severe illness and a fatal outcome of the disease than those that experienced an infection with a seasonal IAV strain. This of course, would be of great concern and is supported by the data obtained in our mouse model. Ideally, seasonal influenza vaccines are used that also induce heterosubtypic immunity [16], [39]. More research is required in this field to define vaccine preparations that not only induce protective immunity against seasonal influenza, but also induce heterosubtypic immunity. With the current pandemic threat caused by A/H5N1 viruses this would be highly desirable [40].

## Materials and Methods

### Viruses

Virus stocks of influenza viruses A/Hong Kong/2/68 (IAV HK/68) and A/Indonesia/5/05 (H5N1) (IAV IND/05) were prepared by infecting confluent Madin-Darby-Canine-Kidney (MDCK) cells. After cytopathologic changes were complete, culture supernatants were cleared by low speed centrifugation and stored at −70°C. Infectious virus titers were determined in MDCK cells as described previously [41].

### Vaccine preparation

Influenza subunit antigen derived from IAV X-31 (H3N2) was essentially prepared as described previously [42]. X-31 is a reassortant vaccine strain of A/Aichi/2/68 and A/PR/8/34, of which the HA and NA resemble that of IAV HK/68 closely. The purity of the subunit preparations was tested by SDS-polyacrylamide gel electrophoresis and the absence of the nucleoprotein and matrix protein of the subunit preparations was tested by western blotting using monoclonal antibodies against the influenza A nucleoprotein and the influenza A matrix protein. The protein concentration was determined using a BCA Protein Assay Kit (Pierce, Rockford, USA).

### Immunization and infection of mice

Female specified pathogens free 6–8 weeks old C57BL/6J (H-2b) mice were purchased from Charles River (Sulzfeld, Germany). Mice were immunized twice with an interval of four weeks intramuscularly (i.m.) in both hind legs in a total volume of 100 µl. Mice (n = 19–40 per group) received PBS (phosphate buffered saline) (Groups 1,3 and 4), 15 µg subunit vaccine with (Groups 2 and 5) or without (Group 6) 1 mg Aluminum hydroxide gel (Alum) (Sigma-Aldrich, Zwijndrecht, The Netherlands) or Alum only (Group 7). Eight days after the second vaccination, four mice of each group were bled and spleens were resected. Four weeks after the second vaccination, mice of groups 2, 3, 6 and 7 were infected intranasally with 5×10^2^ TCID_50_ IAV HK/68 in a volume of 50 µl. Four and twelve days post infection (p.i.), 5–7 mice were bled and lungs and spleens were resected. Four weeks after infection with IAV HK/68, all mice except mice of group 1 were challenged with 2×10^2^ TCID_50_ IAV IND/05. A dose of 2×10^2^ TCID_50_ was used because this was the minimal dose resulting in a lethal infection in >90% mice reproducibly. The day before challenge with IAV IND/05, mice of each group (n = 2–4) were euthanized and lungs and spleens were resected as well as on day four (n = 4–6), seven (n = 2–9) and fourteen (n = 3–8) days after challenge. Vaccinations, intranasal infections, orbital punctures and euthanasia were performed under anesthesia with isoflurane in O_2_. After infection with IAV HK/68 and IAV IND/05, mice were monitored for the presence of clinical signs, including weight loss. All experiments with IAV IND/05 were performed under Biosafety Level 3 conditions. An independent animal ethics committee (DEC consult) approved the experimental protocol before the start of the experiments.

### Serology

Serum samples of mice were collected at various time points during the experiment and tested for the presence of HA-specific antibodies against IAV HK/68 and IAV IND/05 using the hemagglutination inhibition (HI) assay [43] and virus neutralizing (VN) antibodies using the VN assay [44].To determine the titer of antibodies against IAV IND/05 before infection with IAV IND/05, a reverse genetics virus was produced from which the basic cleavage site was removed. Antibody titers obtained with this reverse genetics virus was comparable with that against the wild-type strains (data not shown). Positive control serum specific for IAV HK/68 was obtained by injecting a rabbit with sucrose gradient purified virus [45]. Hyper-immune serum obtained from a swan immunized twice with inactivated H5N2 influenza virus A/Duck/Potsdam/1402/86 (Intervet, Boxmeer, the Netherlands) was used as a positive control against IAV IND/05 [46].

### Lung virus titers

Lungs of mice were snap frozen on dry ice with ethanol and stored at −70°C. Lungs were homogenized with a FastPrep-24 (MP Biomedicals, Eindhoven, The Netherlands) in medium consisting of Hank's balanced salt solution containing 0.5% lactalbumin, 10% glycerol, 200 U/ml penicillin, 200 µg/ml streptomycin, 100 U/ml polymyxin B sulfate, 250 µg/ml gentamycin, and 50 U/ml nystatin (ICN Pharmaceuticals, Zoetermeer, The Netherlands) and centrifuged briefly. Quintuplicate 10-fold serial dilutions of these samples were used to infect MDCK cells as described previously [41]. HA activity of the culture supernatants collected 5 days post inoculation was used as indicator of infection. The titers were calculated according Spearman-Karber [47].

### Flow cytometry of virus-specific CD8+ T cells

#### Peptides and intracellular IFN-γ staining

Single cell suspensions of spleens were prepared as described previously [48]. CD8+ T cell responses after infection were measured by incubation with peptides representing two immunodominant epitopes of IAVs in C57BL/6J mice (H2-b), PA_224–233_ and NP_366–374_ [23], [49]. The peptides of the PA_224–233_ epitope of influenza A virus were manufactured at Eurogentec (Seraing, Belgium), while peptides of the NP_366–374_ epitope were manufactured at Sanquin Research (Amsterdam, The Netherlands). Four hundred thousand splenocytes were cultured for 6 h at 37°C in the presence of 5 µM of either the NP_366–374_ ASNENMDAM (NP_HK_), PA_224–233_ SCLENFRAYV (PA_HK_) peptides derived from IAV HK/68 or the NP_366–374_ ASNENMEVM (NP_IND_) or SSLENFRAYV (PA_IND_) peptides (derived from IAV IND/05) in IMDM (Lonza, Breda, The Netherlands) with 5% FCS and Golgistop (BD). After incubation, cells were o/n stored at 4°C, stained with monoclonal antibody directed to CD3e-PerCP and CD8b.2-FITC, fixate and permeabilized with Cytofix and Cytoperm and stained with monoclonal antibody specific for IFN-γ-PE (all from BD Pharmingen, Alphen a/d Rijn, The Netherlands). Data were acquired using a FACSCalibur and analyzed with Cellquest Pro Software (BD).

#### Tetramerstaining

Splenocytes were washed and stained with mAbs CD3e-PerCP, CD8b.2-FITC (BD Pharmingen, Alphen a/d Rijn, The Netherlands) and either the Phycoerythrin (PE)-labeled H-2Db tetramer with the immunodominant NP_366–374_ epitope derived from IAV X-31 ASNENMETM (Tm_X-31_) or IAV HK/68 ASNENMDAM (Tm_HK_) or the APC labeled tetramer derived from IAV IND/05 NP_366–374_ ASNENMEVM (Tm_IND_). All tetramers were purchased from Sanquin Research, Amsterdam, The Netherlands. Following incubation with tetramers and mAbs for 20 minutes, cells were washed twice and analysed by flow cytometry using a FACSCanto in combination with FACS Diva software (BD).

### Histopathology and immunohistochemistry

After euthanasia, lungs of mice were inflated with 10% neutral buffered formalin. After fixation and embedding in paraffin, lungs were sectioned at 4 µm and tissue sections were examined by staining for hematoxylin and eosin (HE). Using an immunoperoxidase method, sequential slides were also stained with a monoclonal antibody directed against the nucleoprotein of IAV [50].

### Statistical analysis

Data for weight loss after infection, viral load in the lungs, tetramerstaining, and peptide pulsing were analyzed statistically using the two-sided student's T test. Survival was analyzed using the Logrank test. Differences were considered significant at *P*<0.05.
